# Encouraging 5-year olds to attend to landmarks: a way to improve children's wayfinding strategies in a virtual environment

**DOI:** 10.3389/fpsyg.2015.00174

**Published:** 2015-03-12

**Authors:** Jamie Lingwood, Mark Blades, Emily K. Farran, Yannick Courbois, Danielle Matthews

**Affiliations:** ^1^Department of Psychology, University of SheffieldSheffield, UK; ^2^Department of Psychology and Human Development, Institute of EducationLondon, UK; ^3^Laboratoire PSITEC, EDL3, Department of Psychology, Université Lille Nord de FranceLille, France

**Keywords:** virtual environments, wayfinding, landmarks, navigational strategies, route learning

## Abstract

Wayfinding is defined as the ability to learn and remember a route through an environment. Previous researchers have shown that young children have difficulties remembering routes. However, very few researchers have considered how to improve young children's wayfinding abilities. Therefore, we investigated ways to help children increase their wayfinding skills. In two studies, a total of 72 5-year olds were shown a route in a six turn maze in a virtual environment and were then asked to retrace this route by themselves. A unique landmark was positioned at each junction and each junction was made up of two paths: a correct path and an incorrect path. Two different strategies improved route learning performance. In Experiment 1, verbally labeling on-route junction landmarks during the first walk reduced the number of errors and the number of trials to reach a learning criterion when the children retraced the route. In Experiment 2, encouraging children to attend to on-route junction landmarks on the first walk reduced the number of errors when the route was retraced. This was the first study to show that very young children can be taught route learning skills. The implications of our results are discussed.

## Introduction

Psychologists have long been interested in the development of visuospatial abilities (Acredolo, 1977, 1978; Hermer and Spelke, 1994; Nardini et al., 2009; Bullens et al., 2010) and a number of tasks have been used to investigate children's visuospatial abilities such as the reorientation task, the Corsi span task (Corsi, 1972) and the Shapes test (Baddeley et al., 1994). Wayfinding is a specific spatial ability that is measured by investigating the ability to learn and remember a novel route through an environment (Blades, 1991). Wayfinding involves explicit planning and decision making to reach a destination that is beyond one's local surroundings (Montello, 2005). In our everyday lives many of us are able to successfully find our way to familiar or unfamiliar locations (Kitchin and Blades, 2002; Ishikawa and Montello, 2006; Waller and Lippa, 2007; Ishiwaka, 2013). However, very young children find wayfinding difficult (Cornell et al., 1989, 1994; Farran et al., 2010, 2012a) so the current study focused on encouraging young children to attend to landmarks to improve their wayfinding abilities.

Siegel and White (1975) theorized that children learn routes in a series of stages. First, children are able to recognize individual landmarks, then, with more experience children associate the landmarks with specific turns along the route. With even more experience children begin to understand the relationship between the turns and places along the route, and form a “survey” representation of the whole environment. There is some evidence for this theory (e.g., Evans et al., 1981; Cousins et al., 1983; Blades, 1991; Montello, 1998; Ishikawa and Montello, 2006) and evidence that the ability to use landmarks is essential for children's ability to learn and remember novel routes (Acredolo et al., 1975; Cornell et al., 1989, 1992, 1994; Kitchin and Blades, 2002; Jansen-Osmann and Wiedenbauer, 2004; Farran et al., 2012a; Courbois et al., 2013).

Young children rely on the presence of landmarks more than older children (Cohen and Schuepfer, 1980; Jansen-Osmann and Wiedenbauer, 2004), and young children also rely on landmarks being constant because changes in landmark appearance or positioning can disrupt children's ability to use them (Cornell et al., 1994; Heth et al., 1997). Landmarks placed at junctions with an actual or potential change of direction are particularly useful for young children when retracing routes (Allen et al., 1979) and are better memorized than landmarks between junctions (Allen, 1981; Jansen-Osmann and Wiedenbauer, 2004; Janzen and van Turennout, 2004; Schmelter et al., 2009; Farran et al., 2012a). There are also notable differences between how younger and older children use nearby and distant landmarks for wayfinding (Cousins et al., 1983; Cornell et al., 1989, 2001), for instance 12-year olds may scan an environment for distant landmarks, but 6-year olds will focus just on nearby landmarks (Cornell et al., 2001).

Previous researchers have only rarely considered the possibility of training very young children's wayfinding skills so in the two experiments in this paper we investigated whether children could be taught useful wayfinding strategies. Given that young children are particularly dependent on landmarks along a path we developed a training strategy that focused on getting children to attend to the landmarks along a route. Cornell et al. (1989) found that, explicitly pointing out nearby landmarks along a route prevented 6- and 12-year olds from wandering off the route, and in other studies verbally labeling landmarks at junctions reduced the number of route learning errors made by 3-year olds (Darvizeh and Spencer, 1984) and by teenagers (Farran et al., 2010). These studies were conducted in the real world and children walked a route once and then retraced it just once, because walking a route more than twice in a real environment was considered too tiring and stressful for children (Farran et al., 2010). This procedure, although necessary, limits the information that can be gained about children's wayfinding because retracing a route just once does not provide much insight into how children gain route knowledge from progressively more experience as they retrace a route several times. To overcome the limitations of past studies we tested young children in a virtual environment (VE) which provided a safe and flexible way to assess children's wayfinding.

In Experiment 1 we used a desktop (VE). Desktop VEs depict visual and spatial information from a 3D first person perspective (Richardson et al., 1999; Jansen-Osmann, 2002; Montello et al., 2004). VEs provide a safe method for assessing children's wayfinding abilities (Blades, 1997) and VEs allow even very young children the opportunity to experience the same route several times without the physical demands of traveling through a real environment (Broadbent et al., 2014). VEs provide a valid alternative to actual routes, because successful route learning in VEs can transfer to real environments (Ruddle et al., 1997; Montello et al., 2004).

Only one study has investigated landmark training with typically developing children in a VE. Farran et al. (2012a) asked 6- to 9-year olds to learn a route through a VE maze. Children were shown the correct route once by an experimenter and then had to walk the route on two consecutive trials without error. In one condition landmarks along the route were verbally pointed out to children (e.g., “then we go past the bike, then we turn this way at the tree”). In another condition the landmarks along the route were not labeled (e.g., “then we got past here, then we turn this way here”). The maze was then emptied of landmarks, and children walked it again and were asked to recall the identity and location of the landmarks. The verbal labeling had no effect on children's recall. This result suggests that young children may not benefit from training. However, in Farran et al. half the verbally labeled landmarks were at junctions and half were not associated with the junctions but just placed along the path. Children focus more on junction landmarks than on path landmarks (Allen et al., 1979; Allen, 1981; Jansen-Osmann and Wiedenbauer, 2004; Janzen and van Turennout, 2004; Schmelter et al., 2009) and so verbally labeling path landmarks may not have been as helpful as focusing on labeling the junction landmarks. For this reason, in Experiment 1, we concentrated the training solely on the junction landmarks.

In Experiment 1 we asked 5-year olds to learn a route through a VE using a procedure like Farran et al. (2012a). The route had six junctions, each marked by a unique landmark that we will refer to as the junction landmarks. At each junction there was a choice of two directions at each junction: one direction led to the correct path and one direction led to the incorrect path. The incorrect path led to a dead end that was marked by a unique landmark. The landmarks on dead-end paths were never labeled. Children were guided along the correct route once, and were then asked to retrace the route on their own, until they achieved two consecutive error free completions. In condition 1 when children were first shown the route the landmarks were never mentioned, but in condition 2 the landmarks were verbally pointed out by the experimenter (e.g., “we go past the bench then turn this way at the bike”).

Children only begin to explicitly verbalize environmental features like landmarks by about the age of 10 years (Cornell and Heth, 2006), but younger children can represent visual information verbally from an earlier age (Bruner, 1966; Farran et al., 2012b). As noted above, young children are highly reliant on landmarks, and drawing children's attention to landmarks improves route learning in the real world (Darvizeh and Spencer, 1984). Furthermore, previous research has shown that language can play a key role in achieving a goal on a particular task (Cragg and Nation, 2010; Kray and Ferdinand, 2013). For example, verbally labeling the features of working memory tasks benefits young children's ability to maintain a task goal (Kray et al., 2008). Verbally labeling features of a task can also help older children to translate these features into an explicit goal (Chevalier and Blaye, 2009; Lucenet et al., 2014). Therefore, we predicted that in condition 2, when the junction landmarks were verbally labeled by the experimenter, children would perform better than in condition 1 when the junction landmarks were not emphasized by the experimenter.

## Experiment 1

### Method

#### Participants

Forty 5-year olds (*M* = 5.7, SD = 0.27) 18 boys and 22 girls, were recruited from a comprehensive primary school in the UK. Twenty children were randomly allocated to each of condition 1 and 2. Approval for the study was granted by the University of Sheffield, Department of Psychology Ethics Committee.

### Apparatus and materials

#### Virtual environments

Three different mazes in VEs were created using the software program World Viz. VEs were presented to children on a 17-inch Dell laptop that was placed on a desk. Children sat in a chair at the desk 50 cm from the screen. Children navigated through the mazes using the arrow keys on the keyboard.

#### Practice maze

Children were shown a practice maze to familiarize them with using the keyboard to move through a VE. The practice maze was a similar layout to the experimental mazes, but the practice maze did not contain any landmarks.

#### Test mazes

**Figure 1:**
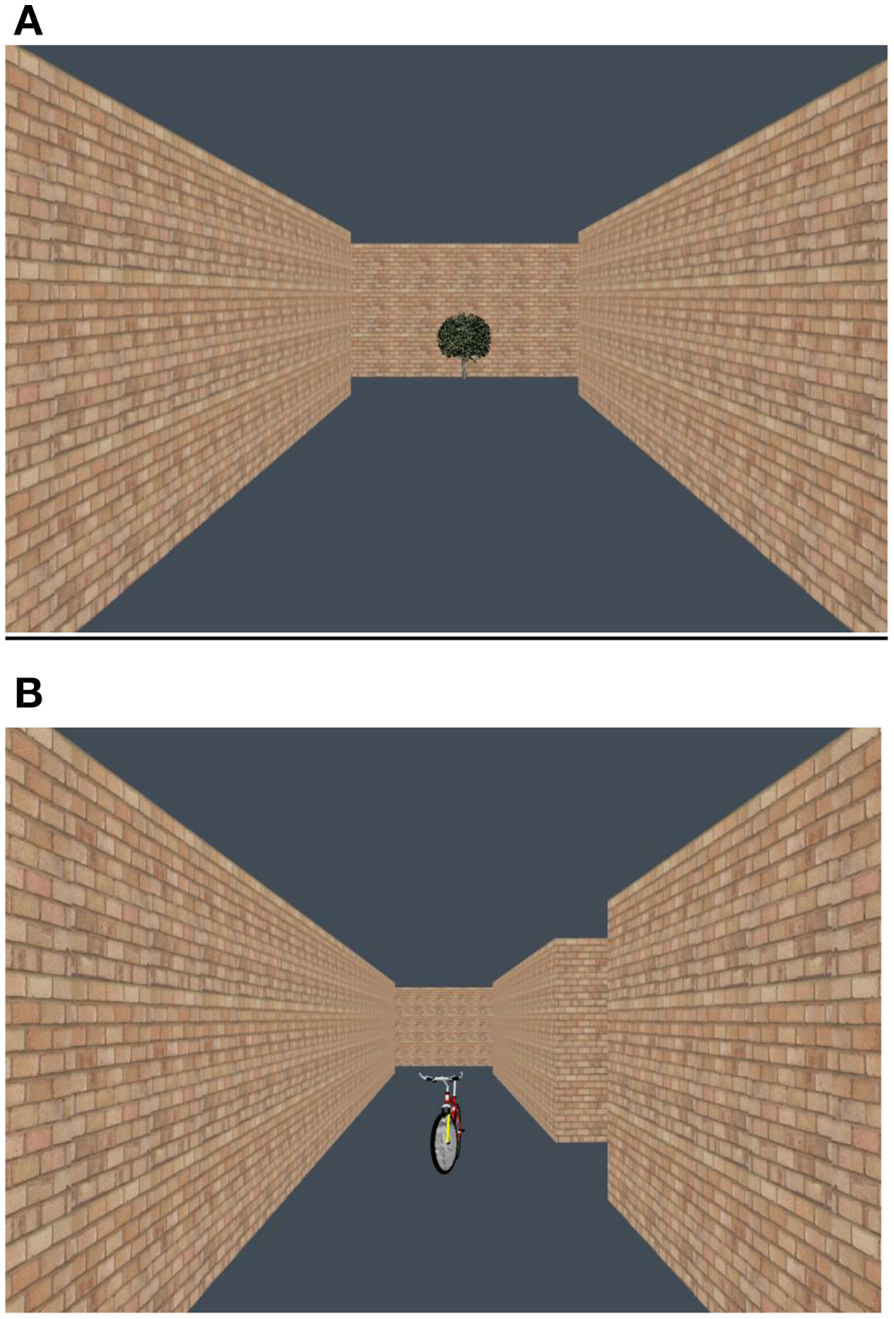
**(A)** Children's view of a “T” junction in Experiment 1. **(B)** Children's view of an “L” junction in Experiment 1.

Two mazes (mazes 1 and 2) were used to test the children. Each maze was a brick wall maze with six junctions. Two of the junctions were “T” shaped junctions and four of the junctions were “L” shaped ones (see Figures 1A,B). Each junction had two paths: a correct path and an incorrect path. Half the landmarks were placed on the correct paths (junction landmarks) and half were placed on incorrect paths (off-route landmarks). As in previous studies all landmarks were placed in the middle of the paths (see Jansen-Osmann and Wiedenbauer, 2004; Farran et al., 2012a,b). Landmarks were placed in the middle of paths in case a child interpreted a landmark that was placed on the left hand side of the path as one that indicated a left turn, or a landmark on the right as one that indicated a right turn.

An incorrect path always ended in a cul-de-sac, but from each junction a cul-de-sac looked like a typical path rather than a dead-end. Therefore, children could not tell that they had made an error until they had actually committed to walking down a chosen path. There were two right, two left and two straight ahead correct choices that were balanced with the same number and types of incorrect choices. All of the path lengths between junctions were equal. A white duck marked the start of the maze and a gray duck marked the end of the maze. When retracing a route from the start a child was told to find the route back to the gray duck. The gray duck provided a salient target that was always the end point of the route, and did not move. When children reached the gray duck, the maze disappeared, indicating the end of a trial.

**Figure 2:**
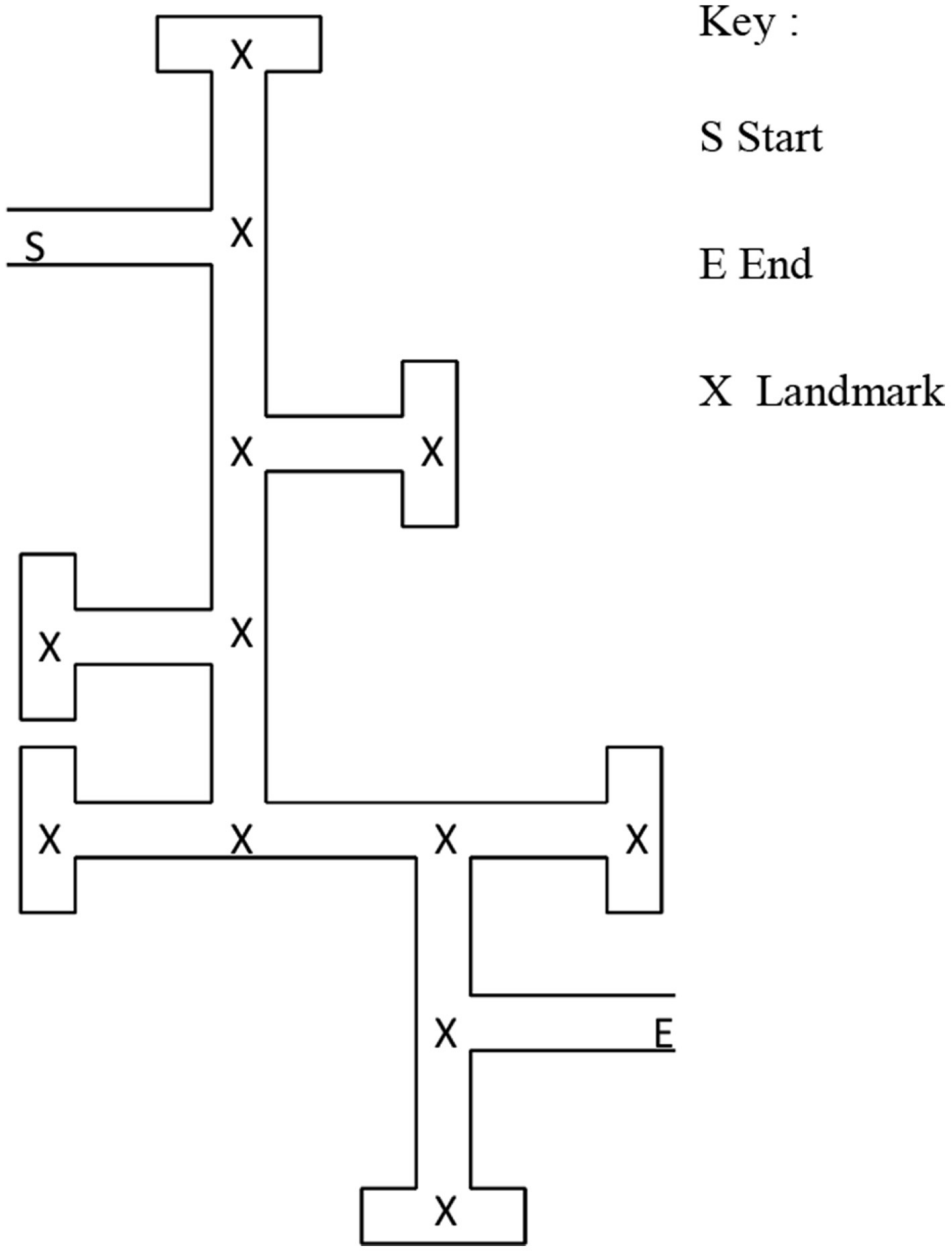
**Plan of maze 1 in Experiment 1**. Participants began at “S” and traveled to “E.” (n.b., maze 2 was the same layout as maze 1 but the start and end points were reversed, so that participants in maze 2 traveled from E to S).

Half the children in each condition were tested with maze 1 and half with maze 2. Maze 2 was the same route as maze 1 but the route was reversed, so that the end point of the maze 1 was the start point for maze 2 (see Figure 2). In both conditions the children were first given a single experience of the correct route, guided by the experimenter. During this initial experience the experimenter guided children by moving forward or turning along the correct route (without looking down any of the incorrect paths). In condition 1, the experimenter simply guided them through the maze e.g., “You go past here, turn this way, and then you turn this way.” In condition 2, the experimenter verbally named each landmark. For example, “You go past the bench, turn this way at the traffic light, and then you turn this way at the bin.” In both conditions, the experimenter did not use any directional language, such as “turn right.” In both conditions, the landmarks were all objects with names that would be familiar to children such as: ball, playground slide, street lamp, umbrella (for a full list see Appendix A and B in Supplementary Data). These items were chosen because they had distinctive names, were easily recognizable, and could be distinguished from each other without difficulty. All the landmarks were static because they did not change position during the experiment. There were six landmarks at junctions on the correct path and six landmarks on the incorrect path. When being shown the route children passed close to the landmarks at junctions, but they were not close to the off-route landmarks.

#### Procedure

Children completed the experiment in a quiet room in their school. Informed consent was obtained from all the children's parents, and all the children were asked if they wanted to take part. None of the children refused to take part.

The child sat at the desk facing a computer and the experimenter sat beside them. The experimenter spent 2 min talking to the child informally to establish rapport. Then the experimenter introduced the task by saying, “This computer has got some mazes on it that we are going to use. First, we're going to practice using the computer to walk around a maze. I'll go first and show you how, and then you can have a turn.” The experimenter then demonstrated how to navigate through the practice maze using the arrow keys. Children were given time to walk around the maze until they were confident about using the arrow keys, at which point the experimenter ended the practice phase by saying, “Well-done, I think you've had enough practice now. Let's have a go at another maze now.”

A child was told: “Now I'm going to show you the way through a new maze. Somewhere in this maze there is a little gray duck to find. I'll show you the way to the gray duck once, and then you can have a go.” The experimenter demonstrated the correct route from the start to the end of the maze, giving verbal instructions that differed according to condition. In condition 1, the experimenter used generic terms such as “You go past here, then you turn this way, and then you turn this way.” In condition 2, the experimenter labeled each landmark, by saying, for example, “You go past the bench, turn this way at the traffic light, and then you turn this way at the bin.” The experimenter never used any directional language, such as “turn right.” At the end of the demonstration, the experimenter said, “Hooray, we've found the duck!,” and the screen went blank.

The child was then asked to retrace the route they had been shown from the start of the maze to the gray duck that was always in the same place. No child ever queried this instruction or asked if the duck had moved. The child navigated through the maze using the arrow keys on the keyboard. The experimenter sat behind the child and traced the exact route the child took on a paper copy of the maze, out of the child's sight. The experimenter timed how long it took the participant to complete the maze.

As in Farran et al.'s (2012a) procedure if, after 5 min a child had not reached the end of a maze on a particular trial, the experimenter ended the trial by saying, “Oops, it looks like you've got a bit lost. Not to worry, let's start back from the beginning, shall we?” Eight children in condition 1 and two children in condition 2 did not reach the end of the maze on one or more trials. A note was made that the trial had been curtailed, and a new trial began. Children did not receive any help in finding their way after the initial demonstration of the correct route. If a child asked which way to go, the experimenter said, “I want *you* to show *me* the way to go. Just try your best.” If a child returned to the start position but thought that they had reached the end, they were told, “You're back at the beginning of the maze now. Let's turn around and try again to remember the way I showed you to the little gray duck.” Nine children in condition 1 and one child in condition 2 went back to the start of the maze on one or more trials.

When the child reached the end of the maze, the experimenter congratulated the child, and asked them to walk the route again from the start. This procedure was repeated until the child had walked the route to a criterion of two consecutive completions without error. At the end of the final trial, the child was thanked, and received a sticker.

If a child had not walked the route with two consecutive completions after 20 min or after eight attempts the experiment was stopped and the children were given a sticker. This was based on the procedure used by Farran et al. (2012a).

### Results

#### Scoring

Successful learning was defined as two consecutive completions of the route without error. To achieve this criterion, children had to walk the route without walking down any incorrect paths on two consecutive learning trials. Walking down an incorrect path was classed as an error. Looking down an incorrect path was not classed as an error. The total number of learning trials to reach criterion excluded the final two perfect trials. For example, if a child made an error on trial 1, but then walked the route without error on trials 2 and 3, they would be scored as having required one trial to reach criterion. A lower score indicated better performance. If a child never achieved the criterion, the number of learning trials was calculated as the number of trials that were completed. For example, if a child completed eight trials within the 20 min cut-off time, but did not complete two consecutive trials without error, they scored eight.

**Figure 3:**
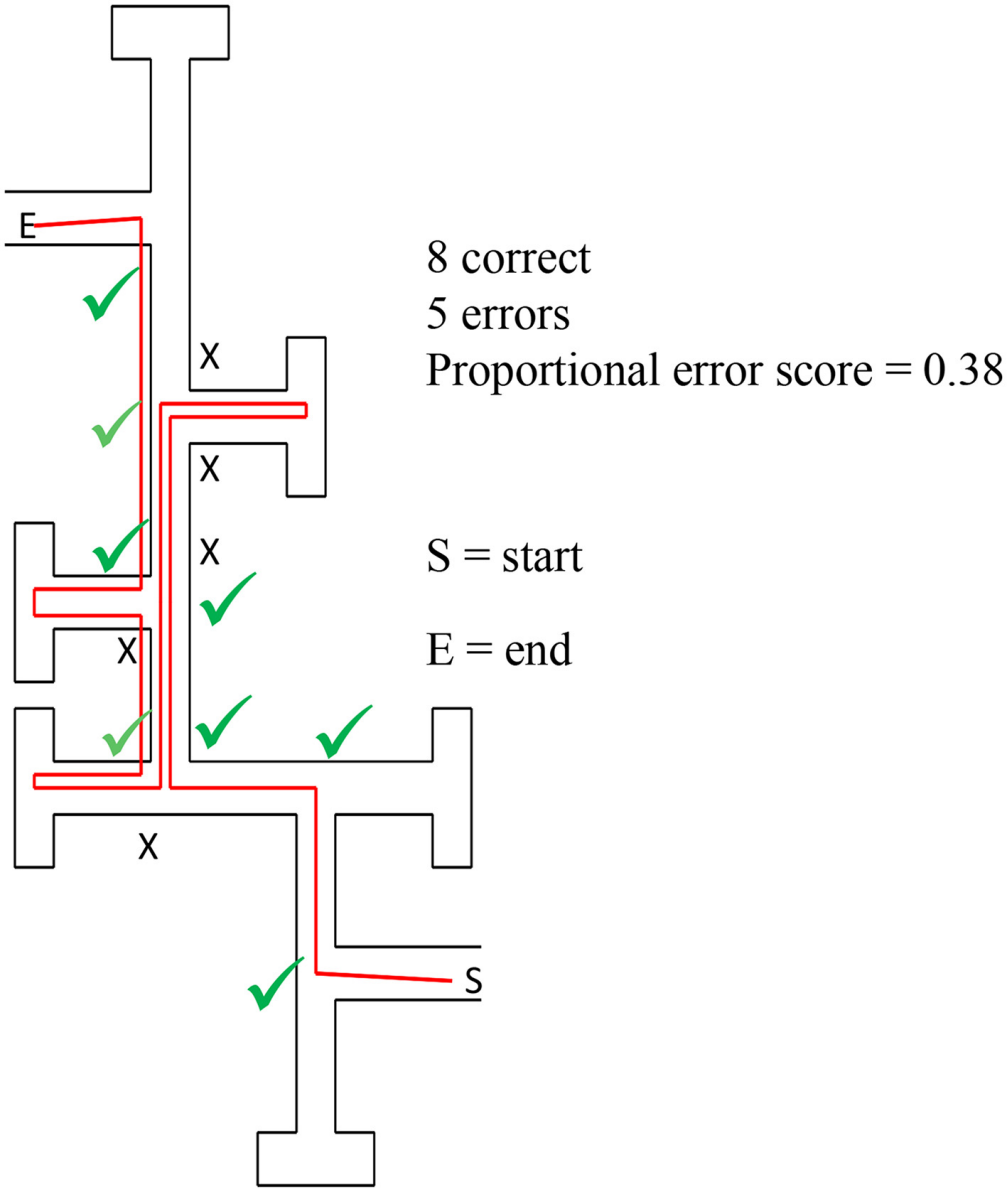
**Example of a route (in red) taken by one child in Experiment 1**. A green tick indicates a correct choice at a junction and a black cross indicates an incorrect choice. This child made eight correct choices and five errors.

Children scored one for every error they made during a trial. On each trial a proportional error score was calculated as the number of errors divided by the number of decisions made. For example, Figure 3 shows the route taken by one child. This child made five errors out of a total of 13 decisions, producing a proportional error score of 0.38. This scoring captured children's wayfinding behavior every time they made a decision, and accounted for occasions when children doubled back and returned to the same junction more than once within a trial. Some children who got lost did not reach the later junctions, so any junctions not reached were also scored as errors at decision points. A mean proportional error score was calculated for each child.

We note that alternative coding criteria produced the same patterns of performance. For example, we coded just the decisions made the first time a participant approached a junction in each trial. Participants scored zero if they chose the correct path or one if they chose the incorrect path and any junctions not reached were counted as errors. Therefore, six indicated the worse performance, and zero indicated perfect performance. When this scoring was compared to the proportional error score (above), there were no differences in the results. Therefore, in the Results section we report only the proportional error scores.

Independent sampled *t*-tests showed that there were no differences between performance on maze 1 and maze 2 for any of the dependent variables, and therefore the two mazes were treated as equivalent in the following analyses. Table 1 shows the mean scores for each condition.

**Table 1:** **Mean scores for number of trials to reach criterion, and mean proportional error score**.

	**Number of trials to reach criterion M (SD)**	**Proportional error score M (SD)**
Condition 1	2.21 (1.58)	0.23 (0.25)
Condition 2	0.89 (1.13)	0.08 (0.13)

*Lower scores indicate better performance*.

#### Number of trials to reach criterion

In condition 1, 14 out of 20 children reached the successful learning criterion of two trials without error and in condition 2, 18 out of children reached that criterion. Of the children who reached criterion, children in condition 2 required fewer trials (*M* = 0.89, SD = 1.13) to reach criterion than children in condition 1 (*M* = 2.21, SD = 0.58), [*t*_(30)_ = 2.77, *p* = 0.01, *d* = 0.96].

#### Proportional errors in the learning phase (all children)

Children in condition 2 (*M* = 0.08, SD = 0.13) had a lower mean proportional error score than children in condition 1 (*M* = 0.23, SD = 0.25), [*t*_(29.41)_ = 2.42, *p* = 0.02, *d* = 0.75].

### Discussion

Experiment 1 showed that when landmarks were verbally labeled by an experimenter children's ability to find their way through a novel route in a VE improved. Children in condition 2 who received verbal labeling training made fewer errors and required fewer trials to reach criterion relative to children in condition 1. In other words, encouraging young children to attend to critical landmarks improved their ability to learn a novel route in a VE.

These results support previous studies which have focused on emphasizing landmarks in real environments (Darvizeh and Spencer, 1984; Cornell et al., 1989; Farran et al., 2010). In our study we found that a large proportion of children (70% in condition 1 and 90% in condition 2) successfully reached criterion. In previous studies children only retraced a route once but by using a VE we were able to ask children to retrace a route a number of times and show that initially emphasizing landmarks had a positive effect on young children's ability to learn a route over a number of trials, and not just on the first time they retraced it.

The only previous study to explore landmark training with typically developing children in a VE by Farran et al. (2012a) who found no effect of verbal labeling which contrasts with the findings from Experiment 1. However, in Farran et al. all the landmarks were labeled for the children. In Experiment 1 only on-route junction landmarks were labeled for children. This may have helped children in Experiment 1 to focus on the landmarks that were the most important ones for learning the route (Jansen-Osmann and Wiedenbauer, 2004; Janzen and van Turennout, 2004; Schmelter et al., 2009).

These findings support previous studies that have shown that verbally labeling task features can benefit children's ability to maintain a goal (Chevalier and Blaye, 2009; Cragg and Nation, 2010; Lucenet et al., 2014). In Experiment 1 children in condition 1 did not spontaneously apply a verbal strategy when retracing the maze. Therefore, verbally labeling landmarks (in condition 2) may have helped children by focusing children's attention on relevant landmarks (on-route junctions) rather than irrelevant landmarks (off-route junctions). This is consistent with previous research within the working memory literature (Towse et al., 2000; Müller et al., 2004). The results of Experiment 1 do not exclude the possibility that children performed better in condition 2 because the landmarks were pointed out to them. In other words, just having the landmarks pointed out to them (without any naming) may have helped children focus on the more important landmarks along the route, and may have done so irrespective of the naming. In the light of the studies noted above it seems likely that naming was an important aspect of drawing children's attention to the most relevant landmarks, but this is an issue that could be investigated in further research, in which landmarks could be pointed out during the first experience of a route, but never explicitly named.

Experiment 1 focused on one wayfinding strategy, and to find out if children could also benefit from other strategies we carried out a second experiment. Previous research has shown that children as young as 5 years can discriminate between landmarks they have seen along a route from other landmarks they did not see on the route (Kirasic et al., 1980). Therefore, we investigated if getting children to discriminate between these two types of landmarks would improve route learning performance.

In Experiment 2 children were walked through a six turn route in a VE by an experimenter, and then they performed a “spot the differences” task (which had nothing to do with wayfinding). In condition 2 the procedure was similar to condition 1, but instead of the spot the differences task children were given a “landmark-pairs” task. For this task children were shown an on-route junction landmark and an off-route landmark for each of the six junctions in the maze that the experimenter had just walked them through. Children were asked to choose which one of the two landmarks the experimenter had walked them past. Having completed either the spot the differences task or the landmark-pairs task all the children were shown a route through a new VE maze before retracing it themselves. We predicted that children in condition 2 who had experienced the landmark-pairs task would learn the new route better than children in condition 1 who did not receive the training.

## Experiment 2

### Method

Thirty-two 5- to 6-year olds (*M* = 5.8, SD = 0.31) participated and were recruited from a comprehensive primary school in the UK. Thirty-two children (16 boys and 16 girls) were randomly allocated to condition 1 or condition 2. Ethical approval was granted by the University of Sheffield, Department of Psychology Ethics Committee.

### Apparatus and materials

#### Practice maze

The same practice maze as in Experiment 1 was used to familiarize children with walking in a VE.

#### Test mazes

The same two VE maze layouts from Experiment 1 (mazes 1 and 2) were used to test participants in Experiment 2. Each maze contained six on-route junction landmarks and six off-route landmarks. On-route landmarks were landmarks placed along the correct path and off-route landmarks were landmarks placed at paths that resulted in a dead-end. The mazes contained landmarks that would be familiar to children e.g., a tree, a car, a bicycle, a bin, a watch. These landmarks were similar to landmarks used in previous studies (Jansen-Osmann and Wiedenbauer, 2004; Farran et al., 2012a,b) and a full list is given in Appendix B in Supplementary Data. All the landmarks were static ones and never changed position during the experiment. As the children would be walking both mazes, maze 2 had landmarks that were different from the ones in maze 1. This was to ensure that children could not use the position of landmarks from maze 1 to help them find their way later in maze 2.

#### Spot the differences task (for condition 1)

In the spot the differences task which was used in condition 1, children had to find five visual differences between two goldfish that were otherwise identical. For example, one of the differences between the two pictures was that in one picture the goldfish had one eye, whereas in the other picture the fish had two eyes. The spot the differences task was not difficult and all the children were able to find all five differences. The task was chosen to occupy the children in condition for approximately the same length of time as the landmark-pairs task took in condition 2, so that children in both conditions retraced the route in maze 1 after the same period of delay. The spot the differences task also removed the possibility that children who were waiting to retrace the route in condition 1 would mentally rehearse the route they had just seen.

#### Landmark-pairs task (for condition 2)

**Figure 4:**
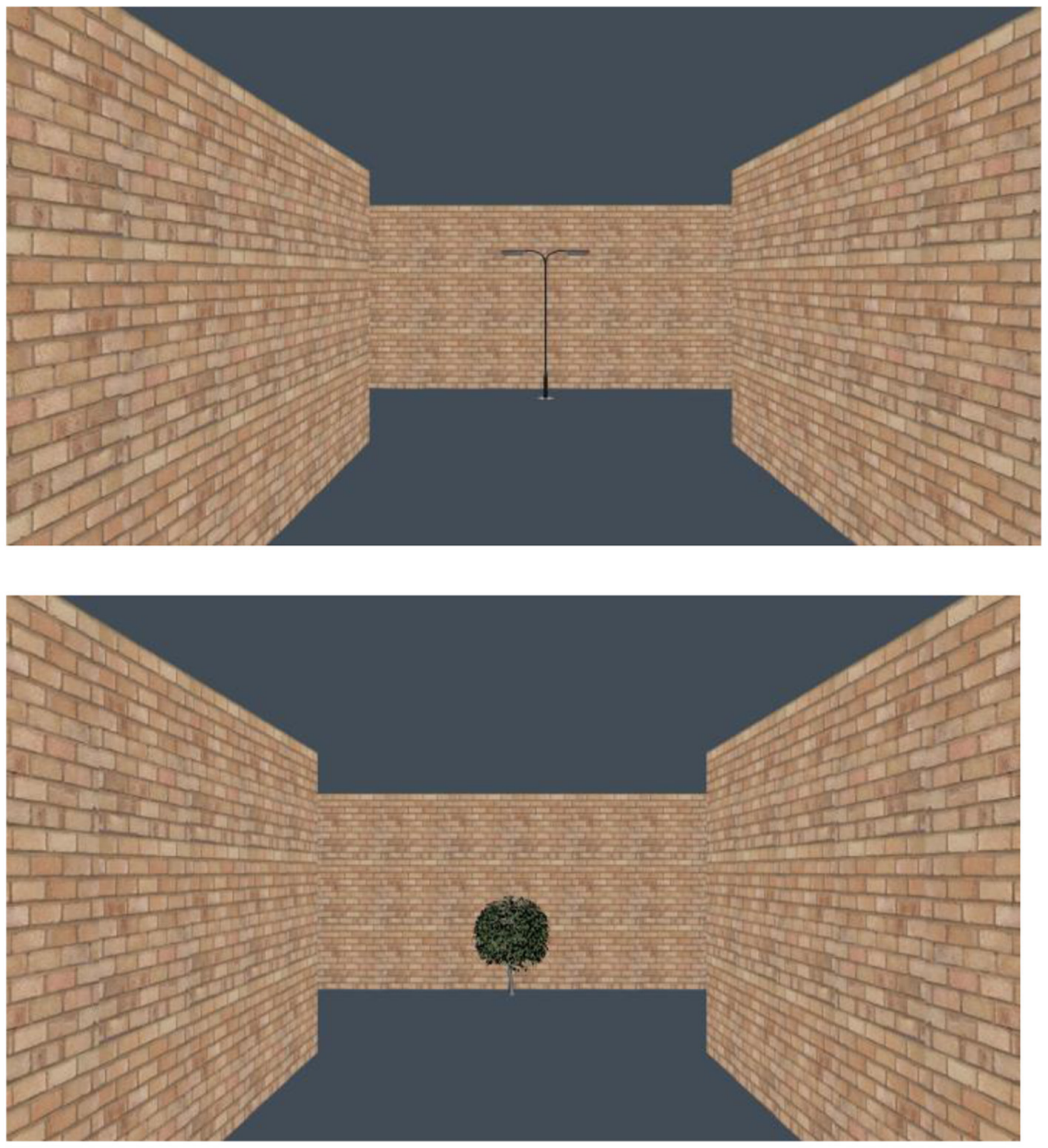
**An example of an “on-route/off-route” landmark pairing used for the landmark-pairs task in condition 2 in Experiment 2**.

Children were shown six pairs of landmarks from maze 1 only, with each pair consisting of an on-route junction landmark and an off-route landmark (see Figure 4). This was repeated for each of the six junctions in the VE in the same order that the experimenter had walked the maze in. For example for junction 1, participants were presented with a tree (on-route junction landmark) and a lamppost (off-route landmark). For each of the landmark pairings participants were asked to choose which landmark the experimenter had walked toward. After the child had responded, the experimenter told the child whether they were correct or not. Children were generally very accurate in this task and identified 94% of the landmark pairs correctly (*M* = 5.63, SD = 0.09). If a child answered incorrectly they were told which landmark was the correct choice.

#### Procedure

Children individually completed the experiment in a quiet room in their school. Informed consent was obtained from all the children's parents, and all the children were asked if they wanted to take part. No child refused to take part. Children were given the same instructions as in Experiment 1 when they were shown the route by the experimenter.

**Figure 5:**
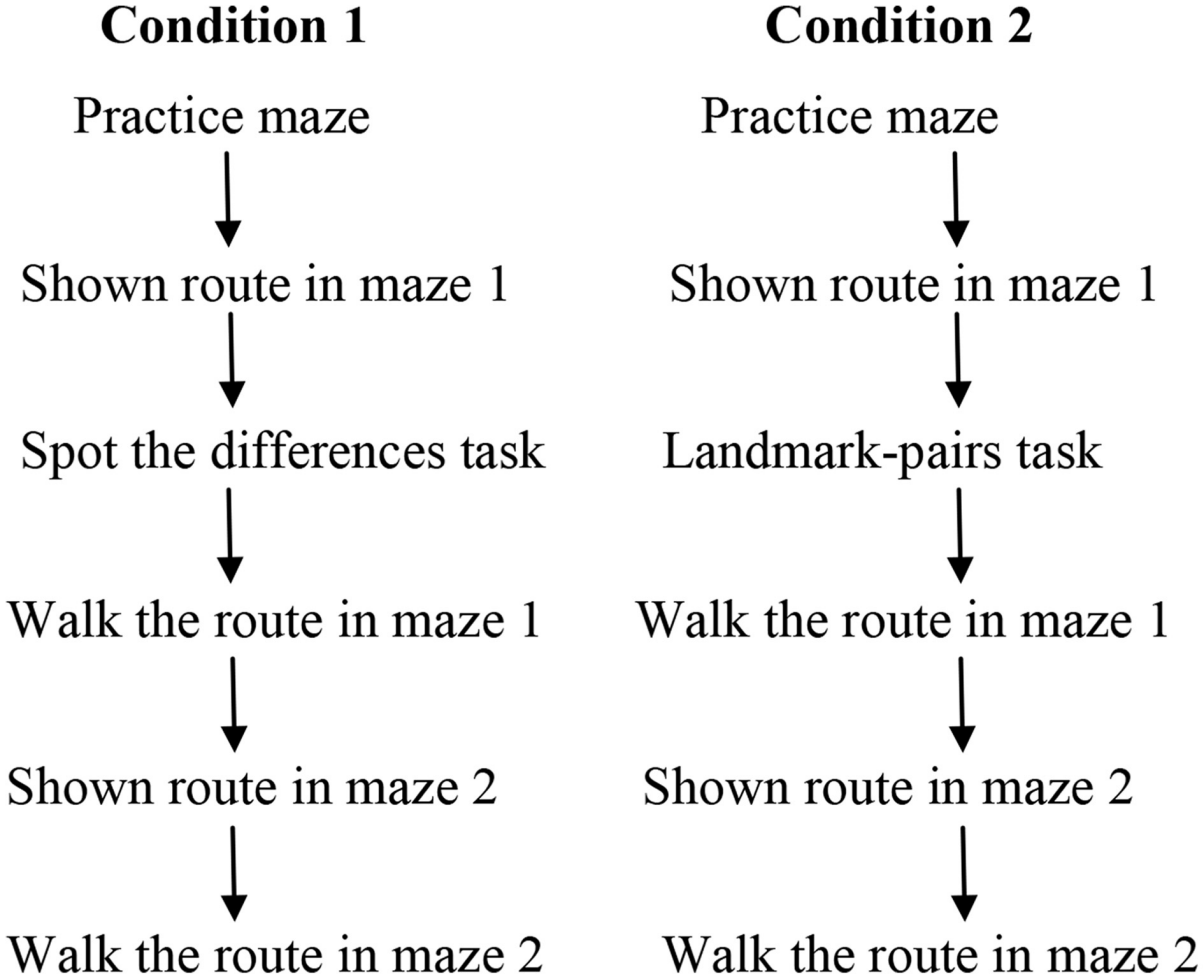
**A flowchart of the procedure used in Experiment 2, condition 1 and condition 2**.

In condition 1 each child was familiarized with moving through a VE by exploring the practice maze (which contained no landmarks). Then the experimenter showed the child the correct route by “walking” the route in maze 1 from start to finish. The experimenter guided the children by moving forward or turning along the correct route (without looking down any of the incorrect paths). After this children completed the spot the differences task. Children then walked maze 1 on their own, once, and their errors were recorded. Following this the experimenter “walked” a new route in maze 2 from start to finish, and finally children attempted to walk the maze 2 route on their own and their errors were noted. In condition 2 the procedure was exactly the same as condition 1 except that children were given the landmark-pairs task instead of the spot the differences task. A flowchart of this procedure from Experiment 2 is shown in Figure 5.

As in Experiment 1, if a child had not reached the end of a maze after 5 min the trial was stopped and a new trial began. Five children in condition 1 and one child in condition 2 did not reach the end of a maze. If a child returned to the start position but thought that they had reached the end, they were told, “You're back at the beginning of the maze now. Let's turn around and try again to remember the way I showed you to the little gray duck.” Five children in condition 1 and one child in condition 2 went back to the start position.

### Results

Children's errors were coded using the same procedure as Experiment 1 to generate a proportional error score. Children's proportional error scores on the two routes they retraced in condition 1 and condition 2 were compared.

When children retraced the first route, in maze 1, there was a marginal difference between the conditions. Children made fewer errors in condition 2 (*M* = 0.18, SD = 0.19) than in condition 1 (*M* = 0.32, SD = 0.24), [*t*_(30)_ = 1.81, *p* = 0.08]. When children retraced the second route, in maze 2, they made significantly fewer errors in condition 2 (*M* = 0.15, SD = 0.16), than in condition 1 (*M* = 0.34, SD = 0.22), [*t*_(29)_ = 2.80, *p* = 0.009, *d* = 0.99]. The latter result showed that in maze 2 children who had earlier been given the landmark-pairs task retraced the route better than children who had not been given the task.

### Discussion

In Experiment 2, children who received the landmark pairs training performed better than the children who did not receive the training. Therefore, the training was an effective way to improve the children's wayfinding performance in the VE maze.

Children made few errors in the landmark-pairs task. This showed that children accurately recalled which landmarks they had passed and which they had not passed in maze 1. This is in line with Kirasic et al. (1980) who found that young children could differentiate between landmarks they had experienced along a route and ones they had not. This also corresponds with other research that has shown children remember more junction landmarks than non-junction landmarks (Allen et al., 1979; Allen, 1981; Jansen-Osmann and Wiedenbauer, 2004; Janzen and van Turennout, 2004; Schmelter et al., 2009; Farran et al., 2012a).

We noted above that the children did very well on the landmark-pairs task, and were almost at ceiling in identifying which landmarks they had walked toward during the first experience of maze 1. We could not directly compare performance on the landmark-pairs task and performance on the actual route task in the mazes, because of the different scoring methods. Although children did very well on the landmark-pairs task, they did less well on retracing the routes in the maze where performance was not at ceiling, especially in condition 1. This might be surprising because if children nearly always recognized the more important landmark in the landmark-pairs task they might have been expected to benefit from this awareness during the route retracing, and make very few errors in the route retracing task as well as in the landmark-pairs task. However, the difference in performance between the landmark maze task and actual route retracing is most likely accounted for by the fact that in the landmark-pairs task the children only had to make a comparative judgment between two simultaneously presented pictures on the screen. In the route retracing task children often only saw one landmark at a time, and saw each landmark from different distances and perspectives as they moved along the route. This reduced the opportunity for making the sort of comparative judgements that were possible in the landmark-pairs task, and may have made the task of retracing the route more difficult than the landmark-pairs task.

The fact that children performed so well in the landmarks-pairs task suggests that the task was not necessarily training them to distinguish between on-route and off-route landmarks, because the children could already do this. More likely the landmark-pairs task reminded children to focus on the landmarks as they walked a route. If so, the landmark-pairs task may have functioned in a similar way to the verbal labeling of on-route landmarks in Experiment 1. We suggest that anything that encourages children to pay attention to appropriate landmarks will benefit their route learning. There may well be other ways to help children focus on the most useful landmarks along a route, and those ways could be investigated in other experiments.

In Experiment 1 the verbal labeling during the initial route experience emphasized the most relevant landmarks which children needed to pay attention to when they retraced that route. In other words, specific training along a route contributed to children's later recall of that route. In Experiment 2 training with the landmark-pairs task helped children retrace the second route in that experiment even though the landmark-pairs task the children had seen earlier was not based on the second route. This suggests that training does not need to be specific to the route children are learning (as in Experiment 1), but will also transfer to learning new routes (as on the second route in Experiment 2).

## General discussion

In the two experiments reported in this paper we found that a small amount of training improved young children's wayfinding performance in a VE maze. Verbally labeling junction landmarks (Experiment 1) and emphasizing junction landmarks (Experiment 2) both had a positive effect on children's performance. These two training measures benefitted children's wayfinding in the VEs we used.

Both studies employed VEs because this allowed us to control the number and the position of landmarks along the routes. Research has shown with adults that tasks in VEs tap into similar cognitive mechanisms as the same tasks in the real world (Richardson et al., 1999), and route learning in a VEs transfers effectively to real world environments (Ruddle et al., 1997; Montello et al., 2004). Darvizeh and Spencer (1984) found that emphasizing landmarks to preschoolers as they walked a novel route to their nursery school did help the children recall the route when they retraced it later. Darvizeh and Spencer's procedure, like the strategies used in Experiments 1 and 2, focused the children's attention on specific landmarks. Therefore, it is possible that the successful strategies we found in the VEs would transfer to real environments, but this would need to be confirmed by carrying out further experiments in the real world.

We recognize that there were differences between the landmarks used in Experiments 1 and 2 and landmarks that are typically found in the real world. For example, in the VEs that were used, landmarks were placed in the middle of the junctions whereas landmarks in the real world are more likely to be placed peripherally. However, previous researchers have found that young children find it difficult to use landmarks that are placed away from paths (Cousins et al., 1983; Cornell et al., 1989, 2001) so placing all of the landmarks centrally ensured that the task would not be too difficult for children, even though this procedure reduced the realism of the VE. In both experiments the landmarks were named by the experimenter during the initial experience of the route. We assumed the names of the landmarks (ball, bin, tree, car, and so on) were within the vocabulary of the children in the study, but in any case the naming procedure was only to draw children's attention to the landmark on the screen, because children did not have to remember the names of the landmarks. In other contexts (e.g., ones with less easily nameable landmarks) it might be more appropriate to let children name the landmarks for themselves, to avoid any risk of the children not relating the adult's label to the landmark being named.

In Experiments 1 and 2 the landmarks were chosen to be easily distinguishable in the maze. They were the same type of objects used as landmarks in previous similar VE experiments (e.g., Farran et al., 2012a; Lingwood et al., 2015). All the landmarks in the mazes were fixed items because they never moved during the course of the experiment. Some of the landmarks (e.g., the tree and the traffic light) were ones that would be fixed objects in a real environment, and others (e.g., the bike and the car) were ones that, in the real world, can move. The former are more reliable landmarks in real environments and, if the children were aware of this, they may have given more attention to the fixed landmarks. Some of the items (e.g., the tree) might be thought of as typical landmarks in the real world, but others (e.g., the umbrella) would not be typical ones. Children may have ignored the less typical items if they considered them less reliable ones, or alternatively they may have given them more attention specifically because they were less typical. However, we do not believe that the nature of the landmarks in the mazes affected the children's performance. Lingwood et al. (2015) used a similar mixture of fixed/movable and typical/atypical landmarks (tree, bench, bike, umbrella, traffic light, and bin) in a VE maze with 6- to 10-year-olds and adults. Lingwood et al. analyzed the number of correct turns at each landmark and found that the type of landmark had no effect on the participants' performance.

In both experiments there was only one landmark at each junction. This is different from most real world contexts where there will usually be a large number of landmarks, including similar ones. On the one hand the strategies we found to be effective in the VEs might not apply so effectively when there are multiple landmarks. On the other hand, such strategies might be particularly appropriate in more complex environments where less experienced wayfinders like children may have difficulty selecting the most appropriate landmarks to encode (Farran et al., 2010, 2012a). How the complexity of an environment affects the choice of wayfinding strategies, in both virtual and real environments, requires more investigation. What Experiments 1 and 2 have demonstrated is that even young children can be taught how to improve their wayfinding, and further research to investigate training in different environments and with other age groups is warranted.

### Conflict of interest statement

The authors declare that the research was conducted in the absence of any commercial or financial relationships that could be construed as a potential conflict of interest.
