# Supplementary material for: Encouraging 5-year olds to attend to landmarks: a way to improve children's wayfinding strategies in a virtual environment
Source: Front Psychol. 2015 Mar 12;6:174. doi: 10.3389/fpsyg.2015.00174 (PMC4357248; doi:10.3389/fpsyg.2015.00174)
Supplement: Supplementary file 1 [file DataSheet1.DOCX]

**Appendix A**

List of landmarks used in Experiment 1, maze 1 and maze 2: Tree, bike, ball, lamppost, car, bin, traffic light, school bus, cow, umbrella, slide, bench.

**Appendix B**

List of landmarks used in Experiment 2.

Maze 1: tree, bike, ball, lamppost, car, bin, traffic light, school bus, cow, umbrella, slide, bench.

Maze 2: watch, torch, tennis racquet, pineapple, crown, hen, dice, flower, teapot, torch, lorry, trumpet.
